# Two-Dimensional Pentagonal Materials with Parabolic Dispersion and High Carrier Mobility

**DOI:** 10.3390/ma17225543

**Published:** 2024-11-13

**Authors:** Xiaofei Shao, Xiaobiao Liu, Xikui Ma

**Affiliations:** 1 School of Mathematics and Physics, University of Science and Technology Beijing, Beijing 100083, China; 2 School of Sciences, Henan Agricultural University, Zhengzhou 450002, China; 3 School of Physics, State Key Laboratory of Crystal Materials, Shandong University, Jinan 250100, China

**Keywords:** ultra-high carrier mobility, planar pentagonal 2D material, parabolic dispersion

## Abstract

Materials with high carrier mobility, represented by graphene, have garnered significant interest. However, the zero band gap arising from linear dispersion cannot achieve an ideal on–off ratio in field-effect transistors (FETs), limiting practical applications in certain fields. In contrast, parabolic dispersion usually exhibits extremely high carrier mobility and an appropriate band gap. In this work, we predicted a planar pentagonal lattice composed entirely of pentagons (namely *penta*-MX_2_ monolayer), where M = Ni, Pd and Pt, X = group V elements. Using first-principles calculations, we demonstrated a parabolic dispersion within this framework, which results in intriguing phenomena, such as a direct band gap (0.551–1.105 eV) and extraordinary high carrier mobility. For *penta*-MX_2_ monolayer, the carrier mobility can attain ~1 × 10^8^ cm^2^ V^−1^ s^−1^ (PBE), surpassing those of black phosphorene, graphene and 2D hexagonal materials. This monolayer also displays anisotropic mechanical properties and significant absorption peaks in the ultraviolet spectrum. Remarkably, 2D *penta*-MX_2_ monolayers are promising for successful experimental exfoliation, particularly when X is a nitrogen element, opening up new possibilities for designing two-dimensional semiconductor materials characterized by high carrier mobility.

## 1. Introduction

Graphene’s intriguing characteristics, including its linear dispersion relation between energy and momentum (the Dirac cone) and its remarkably high carrier mobility [1,2], are rooted in the *π*-conjugation of *p_z_* orbitals within its two-dimensional honeycomb lattice. The tight-binding (TB) model of graphene gives the band spectrum $E\left(\vec{k}\right)=v_{F}\vec{\sigma }\cdot \vec{k}$ in proximity of the *K* point, where $\vec{\sigma }$ is the Pauli matrixes. And the *K* point is the vertex of the Brillouin zone (BZ). Graphene’s Fermi velocity ($v_{F}$) is approximately 1/300 of the velocity of light, which is a significant contributor to its extraordinarily high carrier mobility. This exceptional mobility can reach up to 10^5^ cm^2^ V^−1^ s^−1^ at near-room temperature [3] and low temperature [4], even up to 10^7^ cm^2^ V^−1^ s^−1^ in decoupled graphene measured in a cyclotron resonance (CR) experiment [5]. Despite these advantages, pristine graphene lacks a band gap, which is essential for its use in logic transistors to achieve a high on/off current ratio. A question naturally arises: Can we gain electronic properties superior to graphene, e.g., an opportune bandgap and ultra-high carrier mobility, in other 2D *π*-conjugated systems, such as pentagonal lattice?

Due to the limitations imposed by translational symmetry, lattice structures are unable to have five-fold rotational symmetry, which means that a regular pentagon cannot be the fundamental unit of a two-dimensional lattice. Nonetheless, there are convex pentagons that are capable of tessellating the Euclidean plane in a monohedral arrangement, known as a mathematical challenge of pentagonal tiling. So far, fifteen distinct types of these convex pentagons have been discovered, offering guidance for the development of 2D materials with pentagonal patterns.

Pentagonal graphene was firstly proposed by S. Zhang and collaborators [6], composed of carbon pentagons and known as *penta*-graphene. Unlike planar graphene, *penta*-graphene has a buckled configuration, due to the *sp*^3^-hybridized (four-fold coordinated) carbon atoms in the framework. The presence of *sp*^3^-hybridized carbon atoms breaks the *π*-conjugation of the *p_z_* orbital of *sp*^2^-hybridized carbon atoms, leading to an indirect band gap (~3.25 eV) [6]. However, the indirect band gap is a disadvantage for optoelectronic device applications. Since then, many efforts have been devoted to the design of 2D pentagonal materials. A series of pentagonal materials, such as CN_2_ [7], AlN_2_ [8], B_2_C [9], BP_5_ [10], CdS_2_ [11], SiC_2_ [12,13], and *penta*-silicene [14], were proposed on the basis of first-principles calculations. However, there has been no evidence of π-conjugation properties or the existence of direct band gaps in these works. Additionally, these pentagonal materials are difficult to synthesize in experiments because of the high energy or lack of bulk counterparts.

A pentagonal 2D material, few-layered PdSe_2_ with puckered morphology, has been successfully exfoliated from its bulk crystal counterpart [15]. First-principles calculations indicate that the *penta*-PdSe_2_ monolayer exhibits an indirect band gap of about 1.3 eV, consistent with the optical absorption spectra. A high electron mobility of ~158 cm^2^ V^−1^ s^−1^ was also reported. Recently, L. Liu and collaborators reported the successful synthesis of *penta*-PdTe_2_ monolayer by symmetry-driven epitaxy on Pd(100) substrate, with an indirect bandgap of 1.05 eV [16]. These advances reveal a new approach to achieve pentagonal 2D materials in transition-metal compounds.

In this work, we performed first-principles calculations to introduce a novel class of planar 2D pentagonal materials, namely *penta*-MX_2_, where M = Ni, Pd and Pt, X = group V elements. *Penta*-MX_2_ monolayer is built from relaxing a single layer of MX_2_ crystal with potential for exfoliation. The planar configuration of *penta*-MX_2_ monolayer is perfectly equivalent to the Cairo pentagonal tiling (type 4), which is one of the fifteen known pentagons which can tile the plane monohedrally. An intriguing aspect of *penta*-MX_2_ is the coupling between the *d_π_* (*d_xz/yz_*) orbital of M atoms and the *p_z_* orbitals of X atoms, which creates an extensive *π*-conjugation within the unique framework. This results in exceptional properties that surpass those of graphene. Our first-principles calculations indicate that *penta*-MX_2_ monolayer exhibits a direct band gap and exceptionally high carrier mobilities of ~1 × 10^8^ cm^2^ V^−1^ s^−1^ (PBE), which significantly outperform black phosphorene [17], and rival the carrier mobility of graphene [18]. The intrinsic band gap and ultra-high carrier mobility render *penta*-MX_2_ monolayer an ideal candidate for electronic and optoelectronic applications. Our research further revealed that the natural dispersive bands in the vicinity of the Fermi level and ultra-high carrier mobilities are intrinsic features based on Cairo pentagonal tiling. This discovery not only sheds light on the unique properties of these materials but also paves the way for the development of innovative pentagonal 2D materials with potential applications in various fields.

## 2. Methods and Computational Details

The initial theoretical calculations based on first principles were performed utilizing density functional theory (DFT) with the Vienna ab initio simulation package (VASP) [19,20]. A kinetic energy cutoff of 600 eV was established for the plane wave basis set [21,22], and the interactions between electrons and ions were characterized using the projector augmented wave (PAW) method [23,24]. We employed the Perdew–Burke–Ernzerhof (PBE) exchange-correlation functional within the generalized gradient approximation (GGA) framework for both structural optimization and self-consistent field calculations. For the *penta*-MX_2_ system, we utilized a 7 × 7 × 1 Monkhorst–Pack (MP) [25] *k*-point mesh. The atomic positions and lattice parameters were optimized without imposing any symmetry constraints, continuing the relaxation process until the forces on each atom were reduced to below 10^−3^ eV Å^−1^. The energy convergence criterion was set to 10^−8^ eV/cell. The Methfessel–Paxton [26] smearing method, featuring a smearing width of 0.2 eV, was implemented to handle partial occupancies. To mitigate the self-interaction errors inherent in the PBE functional, a hybrid functional known as Heyd–Scuseria–Ernzerhof (HSE06) [27] was employed. The spin–orbit coupling (SOC) [28] was also considered in our calculations. For the phonon spectrum analysis, density functional perturbation theory (DFPT) and the Phonopy code [29,30], which are compatible with VASP, were utilized. The ab initio molecular dynamics simulations (AIMDS) were conducted in a canonical (NVT) ensemble for the *penta*-MX_2_ material. These simulations employed a Nose–Hoover thermostat to maintain a temperature of 500 K over a duration of 5 picoseconds, with an integration time step of 0.5 femtoseconds.

## 3. Results and Discussion

### 3.1. Structure

The planar pentagon 2D (*P*4*g*) material, namely *penta*-MX_2_ (M = Ni, Pt, Pd, X = N, P) monolayer, is built from relaxing one single layer of bulk MX_2_ crystals. We calculated the cohesive energies for both bulk phases and the pentagon 2D phase using the above-mentioned calculation strategy. Structural optimizations did not take the temperature effects into account [31]. A Monkhorst–Pack (MP) [25] *k*-point grid of dimensions 7 × 7 × 7 was utilized. The lattice parameters obtained from our optimization closely match the experimental data, as detailed in Table 1. The stable bulk phases of PdP_2_, PtN_2_, and PtP_2_ are monoclinic (*C*12/*c*1) [32], orthorhombic (*Pnnm*) [33], and cubic (*Pa*-3) [34,35,36,37,38,39] structures, respectively, all of which have been successfully synthesized in experiments. Our energetic calculations indicate that the orthorhombic (*Pnnm*) and cubic (*Pa*-3) phases of bulk NiN_2_ are nearly energetically equivalent, with the orthorhombic phase slightly more stable by about 0.055 eV/atom.

**Figure 1:**
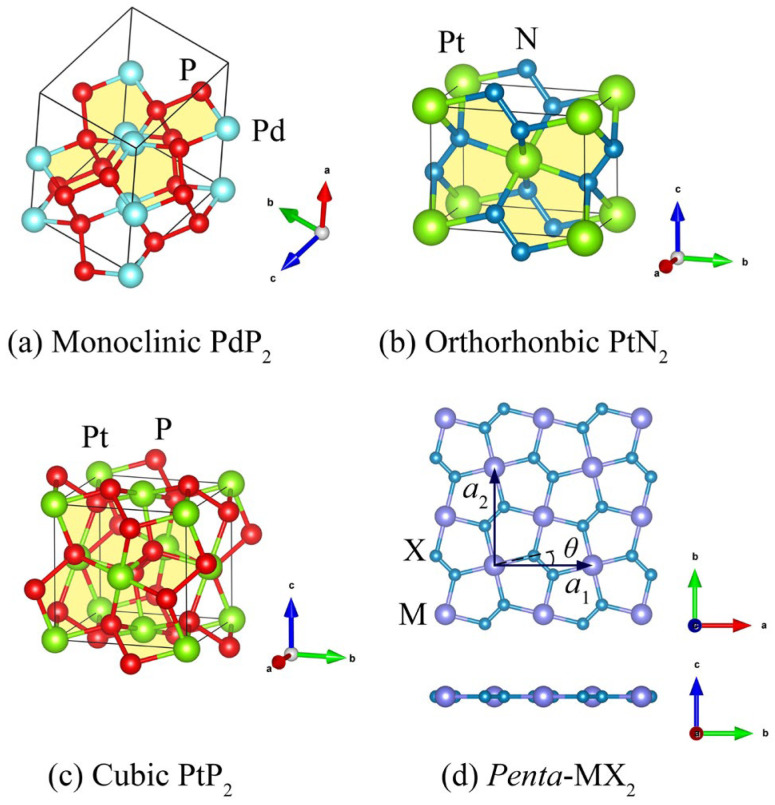
Primitive cell of (**a**) monoclinic PdP_2_ crystal, (**b**) orthorhombic PtN_2_ crystal, and (**c**) cubic PtP_2_ crystal. The (100) plane, (501) plane, and (001) plane are labelled in yellow and exhibit pentagonal lattice features, respectively. (**d**) Top view and side view of *penta*-MX_2_ monolayer.

The atomic arrangements on the (100) plane of monoclinic PdP_2_ crystal, the (501) plane of orthorhombic PtN_2_ crystal, and the (001) plane of cubic PtP_2_ crystal all display buckled pentagonal lattice features, as shown in Figure 1a–c, respectively. Similar to the structural relation between silicene and the bulk silicon crystal, we built a *penta*-MX_2_ monolayer by extracting and relaxing the above single layer of bulk MX_2_ crystal. Once the freestanding single layer had been fully relaxed, it became a planar configuration, as illustrated in Figure 1d and Figure S1. We also examined the interlayer interactions of MX_2_ by relaxing multilayers extracted from three types of bulk NiN_2_ and orthorhombic PtN_2_. We observed no chemical bond formation at least up to five layers, as shown in Figure S2a,c. Conversely, when relaxing bilayers from monoclinic PdP_2_ (Figure S2b) and cubic PtP_2_ (Figure S2d), chemical bonds were observed between adjacent layers. Consequently, the formation of *penta*-MN_2_ monolayers appears to be significantly more feasible than that of *penta*-MP_2_ monolayers.

The synthesis of *penta*-PdTe_2_ monolayer was successfully realized by epitaxial growth on Pd(100) surface [16]. This monolayer formation could be experimentally realized by epitaxial growth on metal M or other substrate materials, as referenced in [40,41]. Here, we propose a possible procedure of experimental growth. (1) Thoroughly clean the surface of M single layer through successive sputtering and annealing in an ultrahigh vacuum (UHV). (2) M atoms on the surface are left with numerous unsaturated bonds, making them highly chemically reactive. Then, X atoms are deposited onto the M monolayer substrate. (3) Annealing at a practical temperature, such as 500 °C, facilitates the formation of *penta*-MX_2_, which may align well with the pentagonal lattice of the underlying M single-layer substrate.

The square planar molecular geometry is a common structural motif for transition metal complexes with a *d*^8^ electron configuration, such as Ni^2+^, Pd^2+^ and Pt^2+^. Interestingly, *penta*-MX_2_ monolayer exhibits a perfect Cairo pentagonal tiling (type 4) with a space group of *P*4*g* [42,43]. This particular instance of type 4 tiling is among the fifteen recognized pentagonal shapes capable of tessellating the plane in a monohedral fashion. Within this pattern, the pentagon exhibits five interior angles of 135° − *θ*, 135° − *θ*, 90°, 90° + 2*θ* and 90°, creating two distinct side lengths. These configurations are depicted in Figure 1d and Figure S1. The values of *θ*, as well as the lengths of the M-X and X-X bonds (representing the two types of sides of the pentagon) are compiled in Table 1. Compared with the bond lengths in bulk MX_2_ crystal, the M-X and X-X bonds are compressed as a result of the quantum-confinement effect. The lattice constants for the square primitive cells of *penta*-NiN_2_, *penta*-PdP_2_, *penta*-PtN_2_, and *penta*-PtP_2_ are 4.538 Å, 5.871 Å, 4.824 Å, and 5.836 Å, respectively. The formation energies of these four *penta*-MX_2_ monolayers relative to the most stable bulk MX_2_ crystal are 0.094 eV/atom, 0.582 eV/atom, 0.021 eV/atom, and 0.699 eV/atom, respectively. For reference, we determined the formation energy of silicene in relation to the bulk silicon crystal, which was approximately 0.744 eV/atom. The formation energies of the *penta*-MX_2_ monolayer are roughly 13%, 78%, 3%, and 94% of that of silicene, respectively. Given that silicene has been successfully synthesized on various substrates, it is plausible to expect the development of *penta*-MX_2_ monolayers in the near future.

### 3.2. Stability

**Figure 2:**
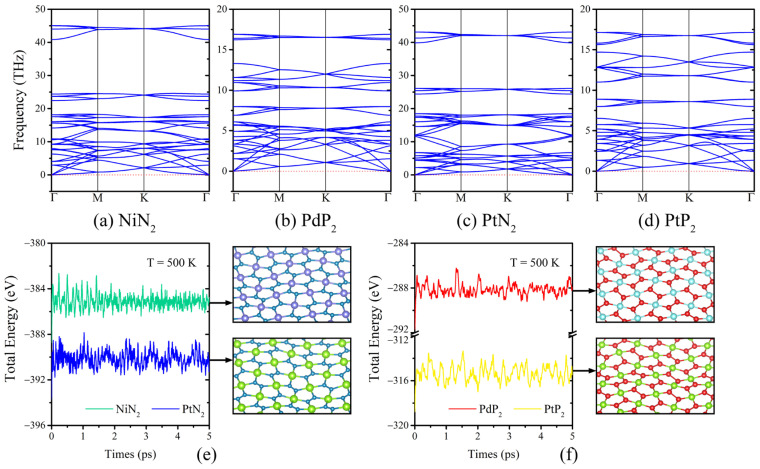
Phonon spectrum of (**a**) *penta*-NiN_2_, (**b**) *penta*-PdP_2_, (**c**) *penta*-PtN_2_, and (**d**) *penta*-PtP_2_ monolayer. Ab initio molecular dynamics simulation (AIMDS) of (**e**) *penta*-NiN_2_ monolayer and *penta*-PtN_2_ monolayer, (**f**) *penta*-PdP_2_ monolayer and *penta*-PtP_2_ monolayer.

We determined the phonon spectrum through first-principles calculations, integrating Density Functional Perturbation Theory (DFPT). A $2\sqrt{2}\times 2\sqrt{2}\times 1$ supercell was utilized, with the force convergence criterion set to 0.01 eV/Å. The calculations incorporated a 3 × 3 × 1 *k*-point mesh. The phonon dispersion for the *penta*-MX_2_ monolayer, traversed along the high-symmetry *k*-points within the Brillouin zone (BZ), is depicted in Figure 2a–d. Modes with imaginary frequencies are absent, even in the long-wavelength region, suggesting the dynamical stability of *penta*-MX_2_ monolayer. 

The lattice vibrations of bulk crystal can propagate in any direction and the three acoustic branches are linear near the *Γ* point. In contrast, the lattice vibrations in two-dimensional materials can only propagate within the monolayer. As shown in Figure 2a–d, one among the three acoustic modes displays a quadratic dispersion near the *Γ* point, which is a characteristic commonly associated with the stability of two-dimensional materials [44]. Similar phonon spectrum and relevant lattice vibrational modes of *penta*-PdTe_2_ monolayer were observed by high-resolution electron energy loss spectroscopy (HREELS) [16], which serves as an experimental validation and significantly strengthens our results.

Employing the Nose–Hoover thermostat with an integration time step of 0.5 femtoseconds, we preformed ab initio molecular dynamics simulations (AIMDS) within a large supercell (3 × 3 × 1) at a temperature of 500 K for a duration of 5 picoseconds to assess the thermal stability, which are illustrated in Figure 2e–f. The findings showed that the system’s total energy settled at a stable point, with no evidence of structural decompositions observed throughout the duration of the simulations. This observation implies that *penta*-MX_2_ monolayer is capable of maintaining its stability at the giving temperature once synthesized. Since the potential energies of molecular dynamics are obtained from first-principles calculations and the demand on computational resources is quite substantial, we only performed the AIMDS at 500 K for 5 ps. Further proofs of thermal stability based on practical applications may refer to higher temperatures and longer timescales.

### 3.3. Electronic Properties

**Figure 3:**
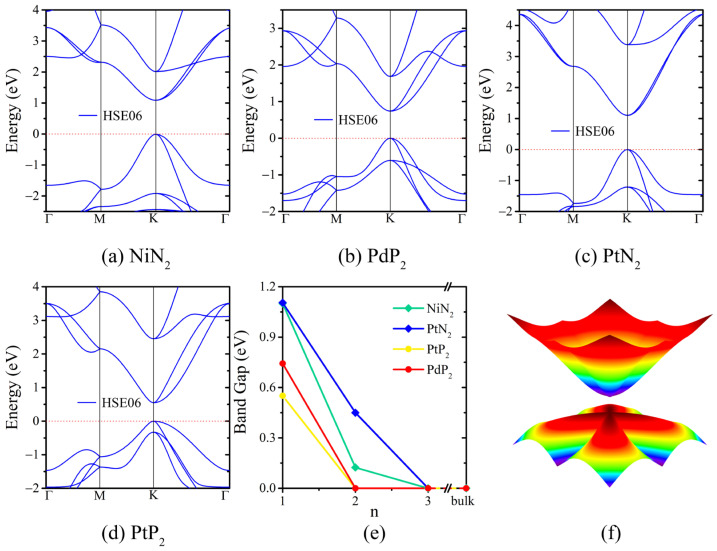
(**a**–**d**) Electronic band structures of *penta*-MX_2_ monolayer, calculated by using the HSE06 functionals. (**e**) Band gap of *penta*-MX_2_ monolayer, bilayer and three layers by using the HSE06 functionals. The number of layers is represented by n. (**f**) Three-dimensional TB band structures of the sixth band (highest VB) and the seventh band (lowest CB) of *penta*-PtP_2_ with TB parameters of *t*_0_ = 2.138, *γ* = 1.717, *γ*′ = 0.57, and *ε* = 0.20 by fitting the HSE06 band structures in (**d**). The 3D energy-momentum dispersion is set in close proximity of the Fermi level calculated in the reciprocal space.

We conducted a study on the electronic properties of *penta*-MX_2_, which were anticipated to outperform those of graphene. The band structure of the *penta*-MX_2_ monolayer, obtained through calculations with the HSE06 functional, is depicted in Figure 3a–d. For comparison, the structures calculated using the PBE functional, both with and without considering spin–orbit coupling (SOC) effect, are presented in Figure S5 and Figure S4, respectively. As listed in Table 2, both functionals indicate a direct band gap at the *K* point, located at (*π/a*, *π/a*), but the band gap values are highly sensitive to the functional used. Specifically, the band gaps are 0.043 eV (PBE) and 1.100 eV (HSE06) for *penta*-NiN_2_, 0.161 eV (PBE) and 0.742 eV (HSE06) for *penta*-PdP_2_, 0.074 eV (PBE) and 1.105 eV (HSE06) for *penta*-PtN_2_, 0.066 eV (PBE) and 0.551 eV (HSE06) for *penta*-PtP_2_. The band gap of *penta*-MX_2_ is narrower than that of phosphorene (1.45 eV) [45]. The band gaps of *penta*-MN_2_ are comparable to that of silicon (~1.17 eV in our current HSE06 calculation and 1.1 eV in reference [46]). Consequently, the band gap of *penta*-MX_2_ monolayer is well suited for applications in long-wavelength optoelectronics. As shown in Figure S5 and Table 2, the SOC effect has a minimal impact on the band dispersion in close proximity to the Fermi level, and thus was not considered in subsequent calculations.

The PBE functional significantly underestimates the band gap compared to the HSE06 functional. The reason for band gap underestimation is DFT-PBE’s inherent inability to account for the structural and energetic changes associated with electron transition [47,48]. When an electron transitions from the HOMO to the LUMO, it causes the energies of all other unoccupied orbitals to increase. Thus, additional energy is demanded to move the electron to the shifted-up LUMO level. The reverse process occurs when the electron returns from the LUMO back to the HOMO [49,50]. Although the hybrid functional (HSE06) is accurate in the description of electronic band structures, it is difficult to converge and usually needs parallel computing, leading the computational cost to be considerably high. Thus, we employed the PBE functional in the calculations of carrier mobilities and mechanical properties.

We further calculated the electronic band gap for *penta*-MX_2_ in the form of bilayer and three layers in AA-stacked pattern. Our findings indicate that the direct band gap at the *K* point remains largely unaffected and is robust against the interlayer interaction, as depicted in Figure 3e and Figure S6. Due to the interlayer coupling, there is a significant reduction in the band gap of the bilayer, compared to that of the monolayer. The band gap reduces to 0.124 eV for *penta*-NiN_2_ bilayer and 0.450 eV for *penta*-PtN_2_ bilayer by employing the HSE06 functional. The robust direct band gap is more advantageous compared to the band gap in Tl_2_O, in which the direct band gap (monolayer) changes to an indirect band gap (bilayer) due to the interlayer interaction [51]. Moreover, electronic band structures of bulk MX_2_ are metallic in nature.

Taking *penta*-PtP_2_ as an example, the electronic states near the Fermi level are primarily contributed by the *p*_z_ orbitals of X atoms and two degenerate *d* orbitals (*d*_xz_/*d*_yz_) of M atoms, as shown in Figure S7. It is noteworthy that there is a significant energy overlap between these *d*-*p* orbitals in proximity of the Fermi level, suggesting a strong conjugation between them. Coupling between the d*_π_*-*p*_z_ orbitals is revealed as *π*-*π* interaction, and facilitates *π*-conjugation across the pentagonal configuration, which accounts for the dispersive parabolic bands in close proximity to the Fermi level. As depicted in Figure S7e, X ions possess a single unpaired electron in the *p*_z_ orbital. M ions exhibit M(II) states, which are characterized by an eight-*d*-electron configuration ($d_{xy}^{↑↓}d_{z^{2}}^{↑↓}d_{xz}^{↑↓}d_{yz}^{↑↓}$). Each *penta*-MX_2_ unit cell has four X atoms and two M atoms, comprising a total of twelve electrons, of which four originate from the *p*_z_ orbitals of the M ions and eight originate from the *d*_xz_ and *d*_yz_ orbitals of M ions. Based on this orbital analysis, we developed a tight-binding (TB) model. In the reciprocal space, the sixth band (highest VB) and the seventh band (lowest CB) display parabolic dispersion and high anisotropy, as illustrated in Figure 3f, Figures S9 and S10. This anisotropy will lead to anisotropic electron transport properties.

### 3.4. Carrier Mobilities

The dispersive dispersion indicates potential ultra-high electron and hole mobilities in *penta*-MX_2_ monolayer. We employed an acoustic phonon-limited scattering model to assess the carrier mobility along *K*-*M* (100) and *K*-*Γ* (110) directions, as depicted in Section S9 in Supplementary Materials. Carrier mobilities of a 2D material can be expressed as $\mu_{2D}=\frac{e\hbar^{3}C_{2D}}{k_{B}Tm_{e}^{*}{\left(E_{l}^{i}\right)}^{2}}$ [18], which is determined by effective masses, deformation potential, elastic modulus, and temperature. In this work, we set temperature at 300 K. The deformation potential and elastic modulus are calculated under proper cell compression and dilatation with a step of 0.5%. In this model, the primary assumptions on scattering mechanisms which limit carrier mobilities are simplified, only taking the scattering of acoustic phonons into account. Other scattering mechanisms, such as optical phonons, Coulomb interactions, impurities, and defects are not considered, tending to overestimate the carrier mobilities compared with experimental values. All the electronic band structures and structural properties necessary for these mobility evaluations were derived from calculations using the PBE functional. The carrier mobilities and relevant parameters of *penta*-MX_2_ exhibit significant anisotropy, as detailed in Table 3.

The DFT calculations using the PBE functional indicated ultra-high carrier mobility mainly in the (100) direction. Within this orientation, *penta*-NiN_2_ demonstrated predominantly high hole mobility of about 1.74 × 10^8^ cm^2^ V^−1^ s^−1^, while *penta*-PtP_2_ exhibited mobilities of 4.31 × 10^7^ cm^2^ V^−1^ s^−1^ for electron and 1.55 × 10^7^ cm^2^ V^−1^ s^−1^ for hole. The hole mobilities of both *penta*-PdP_2_ (1.03 × 10^5^ cm^2^ V^−1^ s^−1^) and *penta*-PtN_2_ (1.74 × 10^5^ cm^2^ V^−1^ s^−1^) in the (100) direction are on par with the theoretical values of graphene monolayer (3.51 × 10^5^ cm^2^ V^−1^ s^−1^), silicene (2.58 × 10^5^ cm^2^ V^−1^ s^−1^), and graphdiyne (2.08 × 10^5^ cm^2^ V^−1^ s^−1^), all of which were assessed employing the identical theoretical approach [18,52]. These ultra-high carrier mobilities are primarily attributed to the ultra-small effective masses, about 0.07 *m*_0_ for *penta*-NiN_2_ and 0.02 *m*_0_ for *penta*-PtP_2_, where *m*_0_ represents the mass of a free electron. Since the bands are converged at the *K* point, we determined the effective masses of hole and electron by fitting the dispersion of VB and CB nearest to the Fermi level in the vicinity of the *K* point. With the exception of *penta*-PtP_2_, all the hole mobilities exceeds the electron mobilities in the (100) direction. Conversely, in the (110) direction, the electron mobilities for all the four *penta*-MX_2_ monolayer surpasses the hole mobilities. And the electron mobility of *penta*-PtN_2_ can also reach 1 × 10^4^ cm^2^ V^−1^ s^−1^ in the (110) direction.

The light and heavy conduction/valence bands are degenerate along the *K*-*M* (100) direction. We determined the mobilities of light carriers along the *K*-*Γ* (110) direction utilizing the PBE functional, focusing on the valence band (light hole) and conduction band (light electron) next-nearest to the Fermi level, as detailed in Table S2. Also, the effective masses of light hole and light electron were obtained by fitting the dispersion of VB and CB next-nearest to the Fermi level in the vicinity of the *K* point. Notably, the mobilities of all these light carriers exceed those of heavy carriers in the same direction. Specifically, for *penta*-PtP_2_, the light hole mobility is determined to be 1.38 × 10^8^ cm^2^ V^−1^ s^−1^ and the electron light mobility reaches 1.33 × 10^9^ cm^2^ V^−1^ s^−1^. These superior mobilities are attributed to the smaller effective masses of *penta*-PtP_2_, evaluated as 0.011 *m*_0_ (light hole) and 0.011 *m*_0_ (light electron).

**Figure 4:**
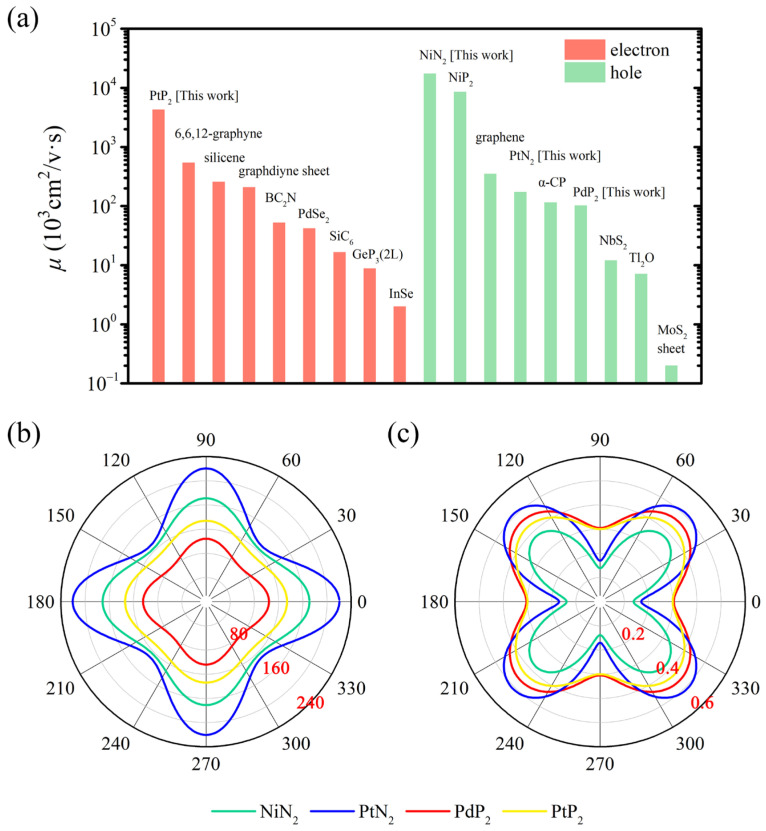
(**a**) Predicted carrier mobilities of typical 2D materials (6, 6, 12-graphyne [53], silicene [52], graphdiyne sheet [18], BC_2_N [54], PdSe_2_ [55], SiC_6_ [56], GeP_3_ (2L) [57], InSe [58], NiP_2_ [42], graphene [18,52], *α*-CP [59], NbS_2_ [60], Tl_2_O [51], MoS_2_ sheet [61]) by employing the acoustic phonon-limited scattering model based on the PBE band structures. For comparison, only the highest value of carrier (electron or hole) mobility of these 2D materials is listed. The unit of mobilities *μ* is 10^3^ cm^2^ V^−^^1^ s^−^^1^. (**b**) Young’s modulus and (**c**) Poisson’s ratio of *penta*-MX_2_ in polar coordinates. The unit of Young’s modulus is GPa·nm.

Plenty of 2D materials have been predicted as high-carrier-mobility materials in previous research [18,51,52,53,54,55,56,57,58,59,60,61], especially in hexagonal lattice. We compared the theoretical values of mobilities in pentagonal lattice (*penta*-MX_2_) with them, as illustrated in Figure 4a. To guarantee a fair comparison, all the listed theoretical values were determined employing the same approach, specifically the PBE functional. The results clearly demonstrate that the carrier mobilities of pentagonal materials stand out among these 2D high-mobility materials, especially much higher than those of hexagonal materials, such as graphene (3.51 × 10^5^ cm^2^ V^−1^ s^−1^) [18,52], 2L graphene (4.64 × 10^5^ cm^2^ V^−1^ s^−1^) [18], silicene (2.58 × 10^5^ cm^2^ V^−1^ s^−1^) [52], BC_2_N (5.26 × 10^4^ cm^2^ V^−1^ s^−1^) [54], PdSe_2_ (4.20 × 10^4^ cm^2^ V^−1^ s^−1^) [55], BC_6_N (12.90 × 10^3^ cm^2^ V^−1^ s^−1^) [62], 2L GeP_3_ (8.84 × 10^3^ cm^2^ V^−1^ s^−1^) [57], Tl_2_O (7.12 × 10^3^ cm^2^ V^−1^ s^−1^) [51], and MoS_2_ sheet (2.00 × 10^2^ cm^2^ V^−1^ s^−1^) [61].

### 3.5. Mechanical Properties

Employing the same elastic theory utilized in the previous calculations of graphene [63], we investigated the mechanical properties of *penta*-MX_2_ monolayer by examining the strain energy in response to the in-plane lattice distortion. In the context of two-dimensional materials, the strain energy per unit area is described by the following equation: $U\left(\varepsilon_{x},\;\varepsilon_{y},\;\varepsilon_{xy}\right)=\frac{1}{2}C_{11}\varepsilon_{x}^{2}+\frac{1}{2}C_{22}\varepsilon_{y}^{2}+C_{12}\varepsilon_{x}\varepsilon_{y}+2C_{66}\varepsilon_{xy}^{2}$ [6], with $\varepsilon_{x}$ and $\varepsilon_{y}$ denoting the uniaxial in-plane strains in *x*- and *y*- axes, respectively. And $\varepsilon_{xy}$ represents the shear strain. The constants $C_{11}$, $C_{22}$, $C_{12}$, and $C_{66}$ correspond to the components of elastic modulus tensor, which are obtained from the second partial derivation of the strain energy with respect to the strains. These elastic constants can be ascertained by fitting the energy curves associated with the uniaxial and equibiaxial strains. Notably, $C_{66}$ is derived by fitting the energy curve under shear strain. The formulas for Young’s modulus and Poisson’s ratio along an arbitrary direction *θ* can be written as $E\left(\theta \right)=\frac{C_{11}C_{22}-C_{12}^{2}}{C_{11}\sin^{4}\theta +C_{22}\cos^{4}\theta +\left(\frac{C_{11}C_{22}-C_{12}^{2}}{C_{66}}-2C_{12}\right)\sin^{2}\theta \cos^{2}\theta }$ and $\nu \left(\theta \right)=\frac{-\left(C_{11}+C_{22}-\frac{C_{11}C_{22}-C_{12}^{2}}{C_{66}}\right)\sin^{2}\theta \cos^{2}\theta +C_{12}\left(\sin^{4}\theta +\cos^{4}\theta \right)}{C_{11}\sin^{4}\theta +C_{22}\cos^{4}\theta +\left(\frac{C_{11}C_{22}-C_{12}^{2}}{C_{66}}-2C_{12}\right)\sin^{2}\theta \cos^{2}\theta }$, respectively [64]. Here, when *θ* = 0° and *θ* = 90°, we can determined the in-plane Young’s modulus and Poisson’s ratio along *x* and *y* directions, respectively, which can be expressed as $E_{x}=\frac{C_{11}C_{22}-C_{12}^{2}}{C_{22}}$, $E_{y}=\frac{C_{11}C_{22}-C_{12}^{2}}{C_{11}}$, $\nu_{xy}=\frac{C_{12}}{C_{22}}$, and $\nu_{yx}=\frac{C_{12}}{C_{11}}$.

The results for the anisotropic Young’s modulus and Poisson’s ratio are depicted in Figure 4b,c and Table 4. These elestic constants satisfy the mechanical stability criterion for 2D materials, $C_{11}\times C_{22}-C_{12}^{2}>0$ and $C_{66}>0$, indicating that all the four *penta*-MX_2_ monolayers are mechanically stable. The in-plane Young’s moduli of the four *penta*-MX_2_ monolayers have been estimated to fall into a wide range (104.019–220.412 GPa·nm). Those values are about 1/3 to 2/3 of that of graphene (with theoretical value of 335 GPa·nm [65,66] and experimental value of 340 ± 50 GPa·nm [67]) and comparable to that of MoS_2_ (123 GPa·nm [68] and 145.82 GPa·nm [69]). For comparison, we give the Young’s moduli of other 2D materials: BN (275.8 GPa·nm) [63], *penta*-graphene (263.8 GPa·nm) [6], *penta*-NiP_2_ (117.42 GPa·nm) [42], SiC_6_ (122.9 GPa·nm and 179.8 GPa·nm) [56], and BC_6_N (308 GPa·nm) [62]. The wide range of Youngs’ modulus in *penta*-MX_2_ monolayer suggests a variable mechanical strength, indicating an untold number of applications such as deformable material and tension activatable substrates.

**Table 4:** Elastic constant of *penta*-MX_2_ monolayer. Young’s modulus and Poisson’s ratio of *penta*-MX_2_ monolayer along *x*- and *y*- axes are listed in the table, in comparison with those of graphene [65,66,67], MoS_2_ [68,69], BN [63], *penta*-graphene [6], *penta*-NiP_2_ [42], SiC_6_ [56], and BC_6_N [62].

*Penta*-MX_2_	Elastic Constant (Gpa·nm)				Young’s Modulus (Gpa·nm)	Poisson’s Ratio	Reference
	$C_{11}$	$C_{22}$	$C_{12}$	$C_{66}$			
*Penta*-NiN_2_	174.280	174.280	24.110	44.814	170.945	0.138	This work
*Penta*-PdP_2_	114.662	114.662	34.933	27.460	104.019	0.305	This work
*Penta*-PtN_2_	226.960	226.960	38.549	43.161	220.412	0.170	This work
*Penta*-PtP_2_	147.082	147.082	44.100	38.119	133.859	0.300	This work
Graphene	/	/	/	/	335	0.16	Theo. [65,66]
					340 ± 50	/	Exp. [67]
MoS_2_	/	/	/	/	123	0.25	Theo. [68]
					145.82	/	Theo. [69]
BN	289.9	289.8	63.7	113.1	275.8	0.220	Theo. [63]
*Penta*-graphene	265	265	−18	/	263.8	−0.068	Theo. [6]
*Penta*-NiP_2_	123.89	123.89	28.33	37.78	117.42	0.229	Theo. [42]
SiC_6_	123	180	−5.2	69.3	122.9 179.8	−0.029 −0.042	Theo. [56]
BC_6_N	/	/	/	/	308	0.179	Theo. [62]

In contrast to *penta*-graphene [6] and SiC_6_ [56], *penta*-MX_2_ monolayer exhibits a positive Possion’s ratio, due to its planar configuration, indicating that *penta*-MX_2_ cannot serve as a nanoauxetic material. The anisotropic electronic and mechanical properties of pentagonal materials are inherent and can preserved under biaxial strain [42,43]. These unique strain-modulated electronic features show *penta*-MX_2_ monolayer to be a versatile 2D material that may find applications in nanomechanics, such as mechanical–electronic devices.

### 3.6. Optical Properties

We use two indices (c and v) to denote the states of the conduction and valence bands, respectively. The cell periodic part of the orbitals at the *k*-point k is represented by $\mu_{ck}$ and the frequency of the incident photon is ω. We have calculated the imaginary component of the complex dielectric function utilizing the following equation [55]: ${\epsilon^{\prime \prime }}_{\alpha \beta \left(\omega \right)}=\frac{4\pi^{2}e^{2}}{\Omega }\times \lim_{q\to 0}\frac{1}{q^{2}}\sum_{c,v,k}2\omega_{k}\delta \left(\varepsilon_{ck}-\varepsilon_{vk}-\omega \right)\times \langle \left.\mu_{ck+eq}\right|\mu_{vk}\rangle {\langle \left.\mu_{ck+eq}\right|\mu_{vk}\rangle }^{*}$. And ${\epsilon^{\prime }}_{\alpha \beta \left(\omega \right)}$ represents the real part of the dielectric function, which can be determined from the imaginary part within the Kramers–Kroning relations. The formula $I\left(\omega \right)=\sqrt{2}\omega {\left[\sqrt{\epsilon^{\prime }{\left(\omega \right)}^{2}+\epsilon^{\prime \prime }{\left(\omega \right)}^{2}}-\epsilon^{\prime }\left(\omega \right)\right]}^{\frac{1}{2}}$ can describe the optical absorption coefficient. $\epsilon^{\prime }$ is the real part and $\epsilon^{\prime \prime }$ is the imaginary part of the complex dielectric function.

**Figure 5:**
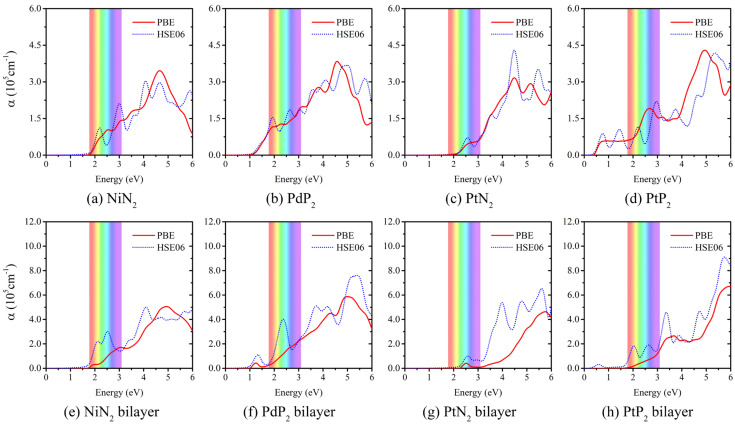
Optical absorption of *penta*-MX_2_ (**a**–**d**) monolayer and (**e**–**h**) bilayer by employing the PBE and HSE06 functionals. The color region represents the visible light region from 1.77 eV (700 nm) to 3.10 eV (400 nm). Scissor operator is applied to align the band gap obtained from the PBE calculations with that from the HSE06 calculations. The multi-peaks observed in the HSE06 data are attributed to the inadequate *k*-point sampling density.

The optical absorption coefficients estimated by employing the HSE06 and PBE functionals are depicted in Figure 5, which corresponds to the optical excitation between the highest VB and the lowest CB near the *K* point. The *penta*-MX_2_ monolayers exhibit noticeable absorption in the important region from 1 eV to 6 eV, which marks the infrared, visible, and ultraviolet (UV) range of the light spectrum, while for AA-stacked bilayers, significant absorption peaks are observed in the UV region with the adsorption to infrared and visible light partially screened. For example, the absorption of near-infrared light in *penta*-PdP_2_ bilayer is partially screened compared to its monolayer, as shown in Figure 5b,f. And pronounced screening in infrared and visible light areas is observed in *penta*-PtP_2_ bilayer (Figure 5h) in contrast to its monolayer (Figure 5d). When X is a nitrogen element (*penta*-NiN_2_ and *penta*-PtN_2_), the absorption coefficients approach a negligible level and are practically zero in the energy region from 1.0 eV to 2.0 eV, as depicted in Figure 5a,c,e,g. These intriguing features make *penta*-MX_2_ promising for the application of UV detectors, for which the screening of infrared and visible light adsorptions is needed. Additionally, as shown in Figure 5e–h, the optical absorption coefficients of *penta*-MX_2_ bilayer reach 10^6^ cm^−1^, which are on par with those of organic perovskite solar cells [70,71].

Notably, in contrast to *penta*-MN_2_, absorption peaks start to emerge in the low-energy region in *penta*-MP_2_ (*penta*-PdP_2_ and *penta*-MP_2_), due to the band gaps of *penta*-MP_2_ being narrower than those of *penta*-MN_2_, as depicted in Figure 5b,d,f,h. Previous studies have demonstrated that the band gaps and energy dispersion of pentagonal materials are sensitive under biaxial strain [42,43]. We expect that the optoelectronic properties of *penta*-MX_2_ can also be strain-induced by external strain or chemical doping.

## 4. Conclusions

The planar 2D pentagonal materials (*penta*-MX_2_ monolayers) are promising for successful experimental exfoliation, particularly when X is the nitrogen element. The configuration of *penta*-MX_2_ monolayer is demonstrated to be stable at room temperature once synthesized. The parabolic dispersion near the Fermi level results in a direct band gap (0.551–1.105 eV by using the HSE06 functional) and remarkably high carrier mobilities of approximately 1 × 10^8^ cm^2^ V^−1^ s^−1^ (PBE). Additionally, *penta*-MX_2_ monolayer displays anisotropic mechanical properties and significant absorption peaks in the ultraviolet spectrum. As expected, the combination of dispersive parabolic bands and ultrahigh carrier mobilities shows *penta*-MX_2_ monolayer to be a promising candidate for applications in high-speed electronics, such as field-effect transistors (FETs).

## Figures and Tables

**Table 1:** Space group, lattice vectors, bond length, and cohesive energies of bulk MX_2_ crystals and 2D *penta*-MX_2_ monolayer. The length is measured in angstroms (Å), and the cohesive energy is expressed in electron volts per atom (eV/atom). For NiN_2_, the energy of the orthorhombic crystal, which is the most energetically stable configuration, is used as the reference value. The other three MX_2_ only have one bulk phase with the energy of monoclinic PdP_2_, orthorhombic PtN_2_, and cubic PtP_2_ taken as references, respectively.

MX_2_	Structure	Space Group	Lattice Vectors (Å)	*d_M-X_* (Å)	*d_X_*_-*X*_ (Å)	Energy (eV/Atom)
NiN_2_	Monoclinic	*C*12*/c*1	Theory: *a* = 5.603, *b* = 4.612, *c* = 4.597, α = γ = 90°, *β* = 126.90°	1.947	1.501	0.419
	Orthorhombic	*Pnnm*	Theory: *a* = 2.846, *b* = 4.628, *c* = 3.766, α = γ = *β* = 90°	2.006	1.261	0.00
	Cubic	*Pa*-3	Theory: *a* = *b* = *c* = 4.621, α = γ = *β* = 90°	2.011	1.278	0.055
	Pentagon 2D	*P*4*g*	Theory: *a* = *b* = 4.538, *θ* = 13.51°	1.881	1.243	0.094
PdP_2_	Monoclinic	*C*12*/c*1	Theory: a = 6.245, b = 5.865, c = 5.885, α = γ = 90°, β = 110.90° Experiment: a = 6.207, b = 5.857, c = 5.874, α = γ = 90°, β = 111.80° [32]	2.347	2.218	0.00
	Pentagon 2D	*P*4*g*	Theory: *a* = *b* = 5.871, *θ* = 18.27°	2.324	2.061	0.582
PtN_2_	Orthorhombic	*Pnnm*	Theory: *a* = 3.188, *b* = 4.858, *c* = 3.748, α = γ = *β* = 90° Experiment: *a* = 3.197, *b* = 4.880, *c* = 3.779, α = γ = *β* = 90° [33]	2.098	1.401	0.00
	Pentagon 2D	*P*4*g*	Theory: *a* = *b* = 4.824, *θ* = 12.77°	2.016	1.261	0.021
PtP_2_	Cubic	*Pa*-3	Theory: *a* = *b* = *c* = 5.713, α = γ = *β* = 90° Experiment: *a* = *b* = *c* = 5.695, α = γ = *β* = 90° [34,35,36,37,38,39]	2.398	2.184	0.00
	Pentagon 2D	*P*4*g*	Theory: *a* = *b* = 5.836, *θ* = 18.54°	2.305	2.073	0.699

**Table 2:** Band gap of *penta*-MX_2_ by using the HSE06 and PBE functionals. The effect of spin–orbit coupling (SOC) effect is tested. 1 L, 2 L, and 3 L represent monolayer, bilayer, and three layers, respectively. All bilayer and three layers are in AA-stacked pattern.

*Penta*-MX_2_	Band Gap (eV)					
	HSE06				PBE	
	w/o SOC				w/o SOC	SOC
	1 L	2 L	3 L	Bulk	1 L	1 L
*Penta*-NiN_2_	1.100	0.124	0	0	0.043	0.050
*Penta*-PdP_2_	0.742	0	0	0	0.161	0.177
*Penta*-PtN_2_	1.105	0.450	0	0	0.074	0.317
*Penta*-PtP_2_	0.551	0	0	0	0.066	0.085

**Table 3:** Theoretical value of carrier mobilities of the *penta*-MX_2_ monolayer and relevant parameters in the phonon-limited scattering model along the [100] and [110] directions by employing the PBE functional. The unit of carrier mobility is 10^3^ cm^2^ V^−1^ s^−1^ and *m*_0_ represents the mass of a free electron.

Carrier Type	[100]				[110]			
	$\frac{m^{*}}{m_{0}}$	$E_{l}$ (eV)	$C_{2D}$ (J·m^−2^)	$\mu_{2D}$ (10^3^ cm^2^ V^−1^ s^−1^)	$\frac{m^{*}}{m_{0}}$	$E_{l}$ (eV)	$C_{2D}$ (J·m^−2^)	$\mu_{2D}$ (10^3^ cm^2^ V^−1^ s^−1^)
***Penta*** **-NiN_2_**								
*e*	0.364	2.455	177.544	4.736	0.361	3.783	144.579	1.651
*h*	0.066	0.223	177.544	17361.935	0.361	5.093	144.579	0.911
***Penta*** **-PdP_2_**								
*e*	0.189	1.433	115.355	33.495	0.419	2.237	101.284	2.458
*h*	0.102	1.520	115.355	102.595	0.235	7.982	101.284	0.615
***Penta*** **-PtN_2_**								
*e*	0.236	1.791	227.797	27.279	0.169	2.868	175.528	15.876
*h*	0.133	1.260	227.797	173.790	0.313	4.421	175.528	1.953
***Penta*** **-PtP_2_**								
*e*	0.021	1.296	147.023	4305.197	0.371	2.137	134.426	4.564
*h*	0.021	2.113	147.023	1551.082	0.798	1.292	134.426	2.691

## Data Availability

The original contributions presented in the study are included in the article/Supplementary Materials, further inquiries can be directed to the corresponding author.

## Supplementary Materials

The following supporting information can be downloaded at: https://www.mdpi.com/article/10.3390/ma17225543/s1, Supplementary materials contains ten sections: (Section S1) Crystal structures of *penta*-MX_2_ monolayer, (Section S2) Crystal structures of bulk MX_2_, (Section S3) Brillouin zone of *penta*-MX_2_ monolayer, (Section S4) Electronic band structures of *penta*-MX_2_ monolayer by using the PBE functionals (w/o SOC), (Section S5) Electronic band structures of *penta*-MX_2_ monolayer by using the PBE functionals (SOC), (Section S6) Electronic band structures of AA-stacked *penta*-MN_2_ (*penta*-NiN_2_ and *penta*-PtN_2_) bilayer by using the HSE06 functional (w/o SOC), (Section S7) Orbital contribution to the bands near the Fermi level, (Section S8) Parabolic bands and tight-binding parameters of *penta*-MX_2_ monolayer, (Section S9) Acoustic phonon-limited scattering model and computational details of carrier mobility, and (Section S10) Light carrier mobility of *penta*-MX_2_ monolayer.
