# Supplementary material for: Two-Dimensional Pentagonal Materials with Parabolic Dispersion and High Carrier Mobility
Source: Materials (Basel). 2024 Nov 13;17(22):5543. doi: 10.3390/ma17225543 (PMC11596016; doi:10.3390/ma17225543)
Supplement: Supplementary file 1 [file materials-17-05543-s001.zip › materials-3281798-supplementary.pdf]

Supplementary Materials for

Two-dimensional pentagonal materials with parabolic dispersion and high carrier mobility

Xiaofei Shao[1,*], Xiaobiao Liu[2], Xikui Ma[3]

[1]*School of Mathematics and Physics, University of Science and Technology Beijing, Beijing 100083, China*

[2]*School of Sciences, Henan Agricultural University, Zhengzhou 450002, China*

[3]*State Key Laboratory of Crystal Materials, School of Physics, Shandong University, Jinan 250100, China*

* Corresponding author, shaoxiaofei@ustb.edu.cn

Contents

S1. Crystal structures of *penta*-MX_2 monolayer

S2. Crystal structures of bulk MX_2

S3. Brillouin zone of *penta*-MX_2 monolayer

S4. Electronic band structures of *penta*-MX_2 monolayer by using the PBE functionals (w/o SOC)

S5. Electronic band structures of *penta*-MX_2 monolayer by using the PBE functionals (SOC)

S6. Electronic band structures of AA-stacked *penta*-MN_2 (*penta*-NiN_2 and *penta*-PtN_2) bilayer by using the HSE06 functional (w/o SOC)

S7. Orbital contribution to the bands near the Fermi level

S8. Parabolic bands and tight-binding parameters of *penta*-MX_2 monolayer

S9. Acoustic phonon-limited scattering model and computational details of carrier mobility

S10. Light carrier mobility of *penta*-MX_2 monolayer

S1. Crystal structures of *penta*-MX_2 monolayer

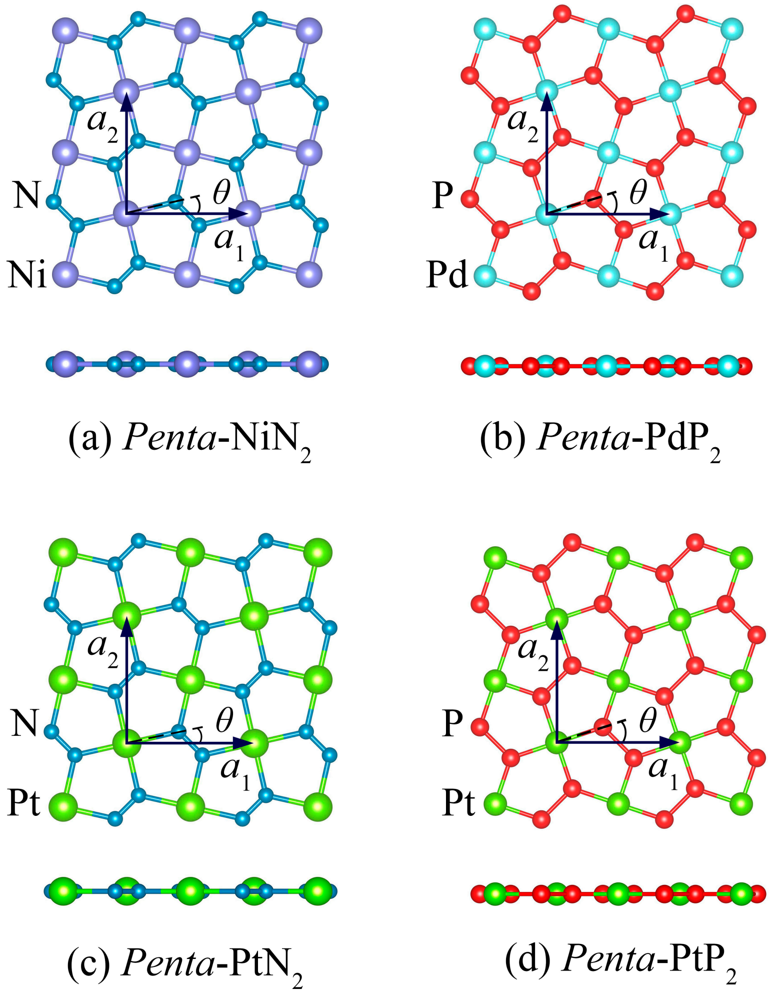

Figure S1. Top view and side view of (a) *penta*-NiN_2 monolayer, (b) *penta*-PdP_2 monolayer, (c) *penta*-PtN_2 monolayer, and (d) *penta*-PtP_2 monolayer.

S2. Crystal structures of bulk MX_2

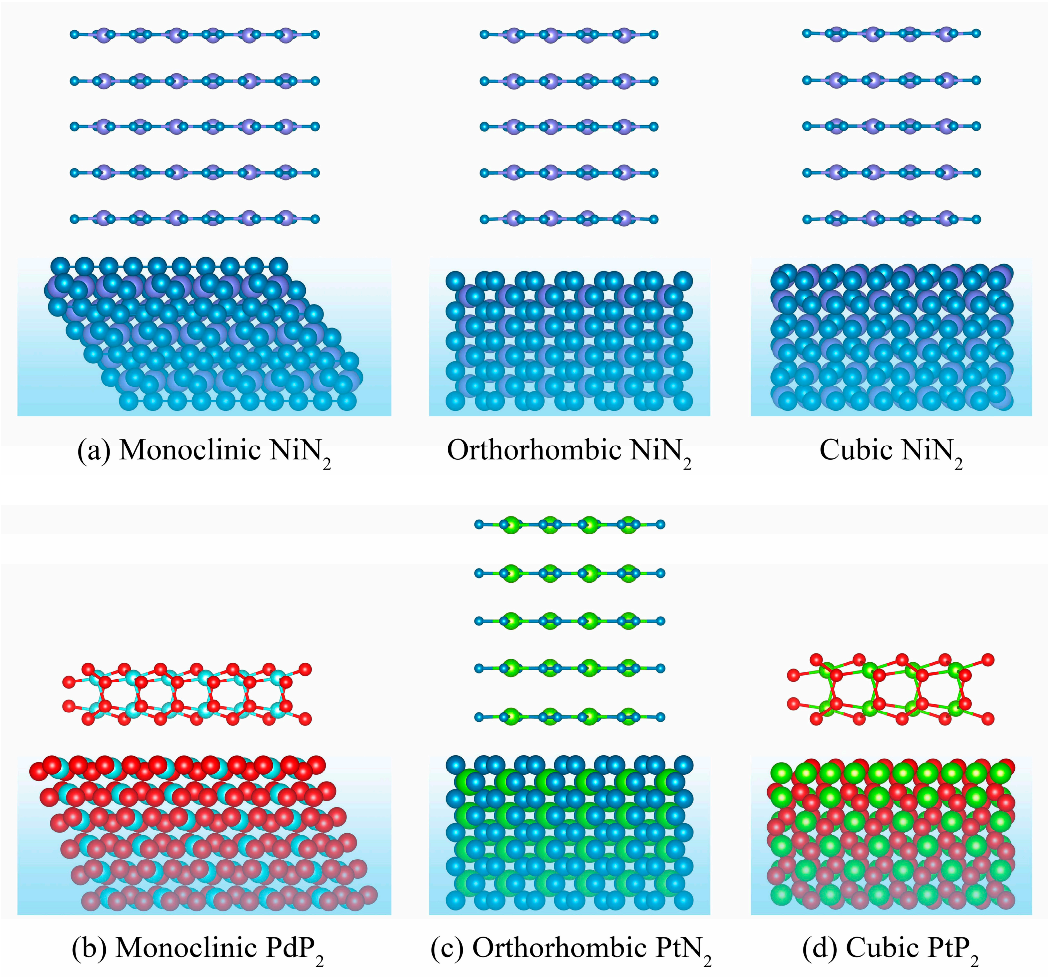

Figure S2. Side view of (a) three kinds of bulk NiN_2 and (c) orthorhombic PtN_2 in contrast with corresponding MX_2 monolayer. For these two structures, there is no forming of chemical bonds at least up to five layers. Side view of (b) monoclinic PdP_2 and (d) cubic PtP_2 in contrast with corresponding MX_2 bilayer. For these two structures, there are chemical bonds between adjacent layers.

S3. Brillouin zone of *penta*-MX_2 monolayer

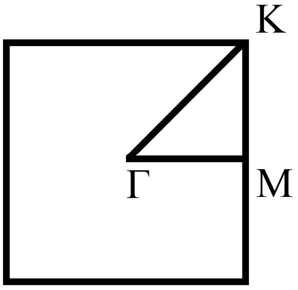

Figure S3. Brillouin zone (BZ) and high symmetry *k*-points of *penta*-MX_2 monolayer. The coordinates are Γ(0, 0, 0), M (0.5, 0, 0), and K (0.5, 0.5, 0).

S4. Electronic band structures of *penta*-MX_2 monolayer by using the PBE functionals (w/o SOC)

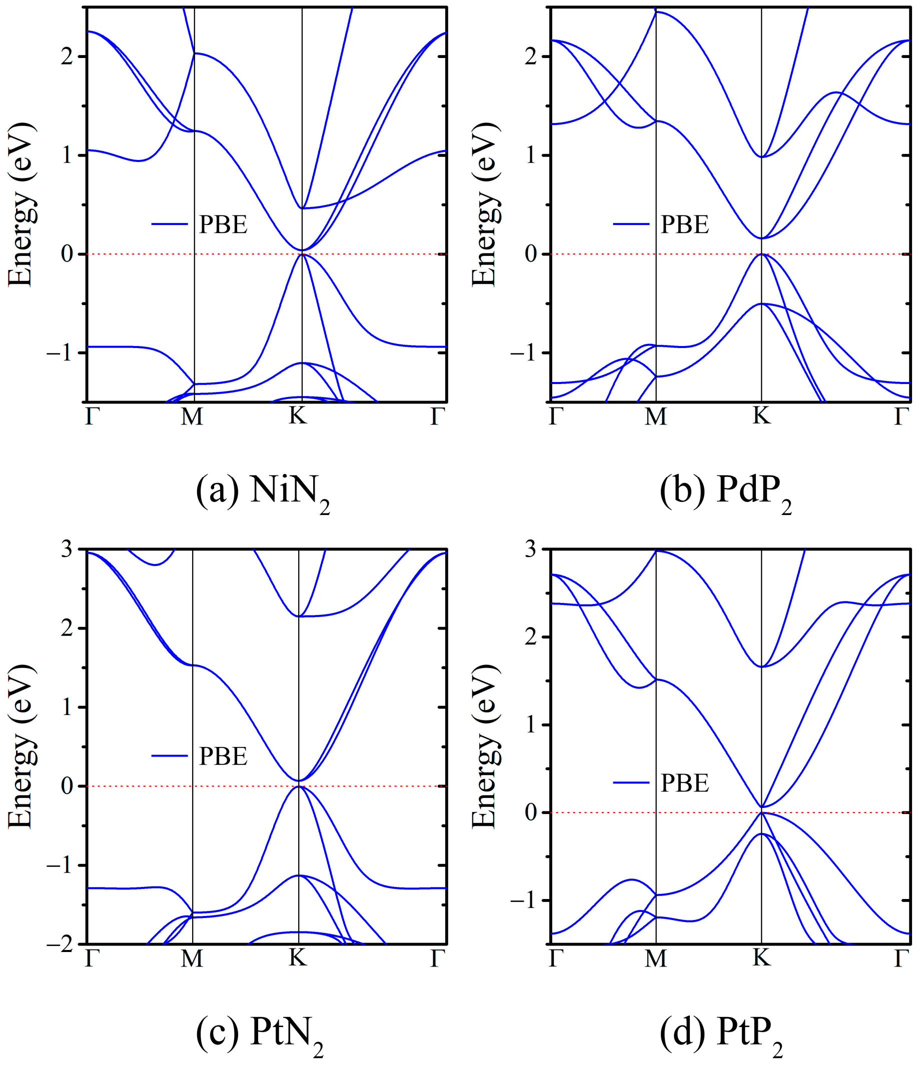

Figure S4. Electronic band structures of (a) *penta*-NiN_2, (b) *penta*-PdP_2, (c) *penta*-PtN_2, and (d) *penta*-PtP_2 monolayer by employing the PBE functionals without taking the spin–orbit coupling (w/o SOC) into account. The energy at the VBM is set to zero.

S5. Electronic band structures of *penta*-MX_2 monolayer by using the PBE functionals (SOC)

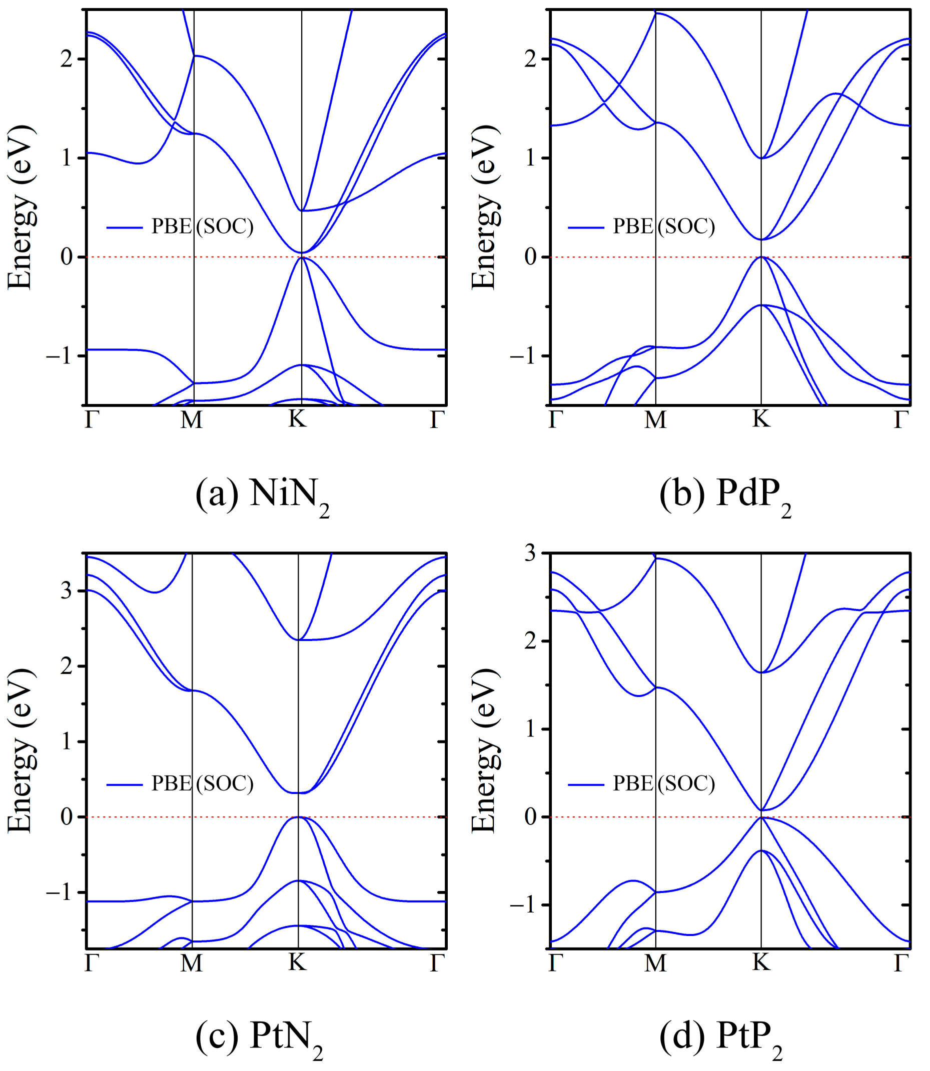

Figure S5. Electronic band structures of (a) *penta*-NiN_2, (b) *penta*-PdP_2, (c) *penta*-PtN_2, and (d) *penta*-PtP_2 monolayer by employing the PBE functionals with the spin–orbit coupling (SOC) effect taken into account. The energy at the VBM is set to zero.

S6. Electronic band structures of AA-stacked *penta*-MN_2 (*penta*-NiN_2 and *penta*-PtN_2) bilayer by using the HSE06 functional (w/o SOC)

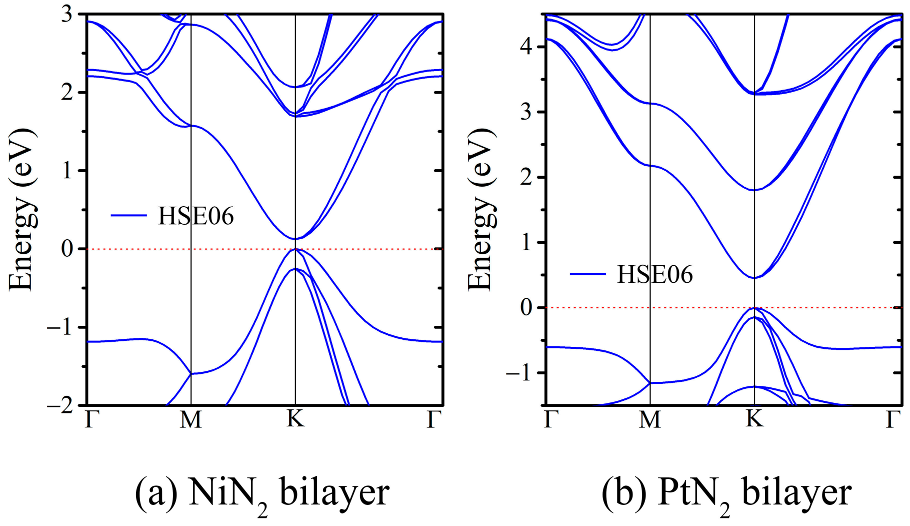

Figure S6. Electronic band structures of (a) *penta*-NiN_2 and (b) *penta*-PtN_2 AA-stacked bilayers by using the HSE06 functionals without taking spin–orbit coupling (SOC) effect into account. The energy at the VBM is set to zero.

S7. Orbital contribution to the bands near the Fermi level

The origins of the dispersive bands can be revealed by the orbital resolved electronic density of states (PDOS). We took *penta*-PtP_2 as an example and projected the electronic density of states (PDOS) onto the atomic orbitals of Pt and P atoms, as shown in Figure S7(a) and Figure S7(b), respectively. Clearly, the electronic states in the proximity of the Fermi level arise mainly from the d_{xz}/d_{yz} orbitals of Pt atoms and p_z orbitals of P atoms. According to the crystal field theory, in a crystal-field environment with a square symmetry, the 5*d* orbitals of the Pt^{2+} split into d_{z^2}, d_{xy}, $d_{x^2-y^2}$ orbitals and a doubly-degenerate $d_\pi\left(d_{xz}+d_{yz}\right)$ orbitals, as shown in Figure S7(e). For *penta*-NiN_2, *penta*-PdP_2, *penta*-PtN_2, and *penta*-PtP_2, the inequivalence of the d_{xz} and d_{yz} orbitals in the orbital resolved electronic density of states (PDOS) is attributed to the angle (θ = 13.51°, 18.27°, 12.77°, and 18.54°) between the M-X bond and *x*- (or *y*-) direction, as shown in Figure 1, Figure S1 and Table 1. Notably, the energy overleapt between d_{xz}/d_{yz} orbitals of M atoms and p_z orbital of X atoms overleaps near the Fermi level, which implies the strong coupling between them. The *π*-*π* interaction between the p_z-d_π orbitals leads *π*-conjugation throughout the pentagonal framework, which is responsible for the dispersive parabolic bands in the proximity of the Fermi level. These features are further confirmed by the charge density of the electronic wavefunctions of the conduction band minimum (CBM) and valence band maximum (VBM), as shown in Figure S7(c) and Figure S7(d), respectively.

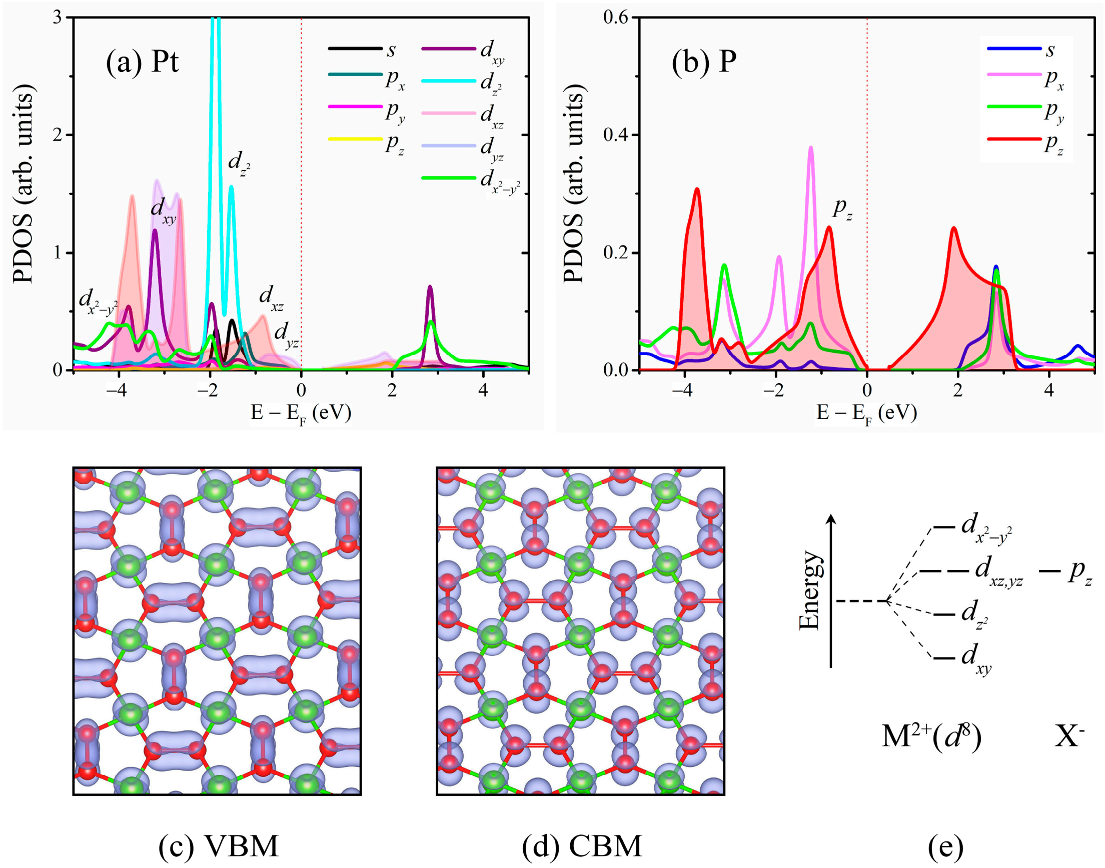

Figure S7. Orbital-resolved electron density of states (PDOS) projected onto (a) Pt and (b) P atoms. The energy at the VBM is set to zero. The isosurfaces of the Kohn-Sham electron wave functions of the (c) VBM and (d) CBM of *penta*-PtP_2. (e) Schematic representation of the *d*-orbital splitting and *p*-*d* π conjugation.

S8. Parabolic bands and tight-binding parameters of *penta*-MX2 monolayer

The geometry of the 2D Cairo lattice originates from a special pentagon that can tile a Euclid plane monohedrally. The pentagon unit has the inner angles of $135^{\circ}-\theta$, $135^{\circ}-\theta$, 90º, $90^{\circ}+2\theta$, and 90º, forming a square lattice with a point group of C_{4h}. The length of the basis vectors is $a=2a_0(\alpha+\beta)$ with $\alpha=\sin\theta$ and $\beta=\cos\theta$. We considered a 2D Cairo lattice of MX_2 (*penta*-MX_2 monolayer) by means of first-principles calculations. M and X atoms reside at the fourfold-coordinated and threefold-coordinated sites, respectively, as shown in Figure S8.

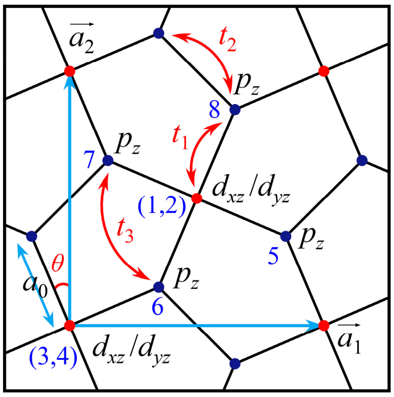

Figure S8. Schematic representation of the TB model of 2D Cairo lattice.

To reveal the origins of the dispersive parabolic bands in the proximity of the Fermi level, we considered a tight-binding (TB) model which contains two orthorhombic d_{xz} and d_{yz} orbitals (d_{π}) at the fourfold-coordinated vertex and one p_z orbital at the threefold-coordinated vertex, as shown in Figure S8. The onsite energy difference between d and p orbitals is represented by ε. The electron hopping between the

nearest-neighbor sites with the amplitudes of t_1 (d - p) and t_2 (p - p) and the p - p hopping between the next-nearest-neighbor sites with the amplitude of t_3 are included. Setting $a_0 = 1.0$, $V_{dp\pi} = -t_0\left(t_0 > 0\right)$, $t_2 = -\gamma t_0$, and $t_3 = -\gamma' t_0$, we got the TB Hamiltonian of the Cairo lattice:

$$H = \begin{pmatrix} H_{dd} & H_{dp} \\ H_{dp}^{*} & H_{pp} \end{pmatrix},$$

with

$$H_{dd} = \begin{pmatrix} \varepsilon & 0 & 0 & 0 \\ 0 & \varepsilon & 0 & 0 \\ 0 & 0 & \varepsilon & 0 \\ 0 & 0 & 0 & \varepsilon \end{pmatrix}, \quad H_{dp} = -t_0 \begin{pmatrix} -\beta S_{15} & \alpha S_{16} & \beta S_{15}^{*} & -\alpha S_{16}^{*} \\ \alpha S_{15} & \beta S_{16} & -\alpha S_{15}^{*} & -\beta S_{16}^{*} \\ \alpha S_{35} & -\beta S_{36} & -\alpha S_{35}^{*} & \beta S_{36}^{*} \\ -\beta S_{35} & -\alpha S_{36} & \beta S_{35}^{*} & \alpha S_{36}^{*} \end{pmatrix},$$

$$H_{pp} = -t_0 \begin{pmatrix} 0 & \gamma' S_{56} & \gamma S_{57} & \gamma' S_{58} \\ \gamma' S_{56}^{*} & 0 & \gamma' S_{58} & \gamma S_{68} \\ \gamma S_{57}^{*} & \gamma' S_{58}^{*} & 0 & \gamma' S_{56}^{*} \\ \gamma' S_{58}^{*} & \gamma S_{68}^{*} & \gamma' S_{56} & 0 \end{pmatrix},$$

$S_{15}{=}e^{i(x_2-y_1)}$, $S_{16}{=}e^{-i(x_1+y_2)}$, $S_{35}{=}e^{-i(x_1-y_2)}$, $S_{36}{=}e^{i(x_2+y_1)}$, $S_{56}{=}2\cos\left(x_1+x_2\right)e^{i(y_1-y_2)}$, $S_{57}{=}e^{2i(x_1+y_1)}$, $S_{58}{=}2\cos\left(y_1+y_2\right)e^{i(x_1-x_2)}$, $S_{68}{=}e^{2i(x_1-y_1)}$, $x_1 = \alpha k_x$, $x_2 = \beta k_x$, $y_1 = \alpha k_y$, and $y_2 = \beta k_y$. The TB Hamiltonian gives eight bands indexed from 1 to 8 in the increasing order of energy, which are independent of θ due to the unitary transformation between the Hamiltonians of different θ.

Penta-MX_2	Fitting the DFT-HSE06 band structures			
	t_0	γ	γ'	ε
Penta-NiN_2	1.810	1.992	0.80	0.20
Penta-PdP_2	1.644	1.869	0.20	0.20
Penta-PtN_2	2.445	1.869	0.78	0.20
Penta-PtP_2	2.138	1.717	0.57	0.20
***Penta*-MX_2**	**Fitting the DFT-PBE band structures**			
	t_0	γ	γ'	ε
Penta-NiN_2	1.530	1.540	0.75	0.20
Penta-PdP_2	1.414	1.606	0.16	0.20
Penta-PtN_2	2.007	1.546	0.74	0.20
Penta-PtP_2	1.841	1.545	0.54	0.20

Table S1. TB parameters of *penta*-MX_2 obtained by fitting the DFT-HSE06 and DFT-PBE band structures. ε represents the onsite energy difference between d and p orbitals. t_1 (d - p) and t_2 (p - p) represent the amplitudes of electron hopping between the nearest-neighbor sites and t_3 represents that of p - p hopping between the next-nearest-neighbor sites.

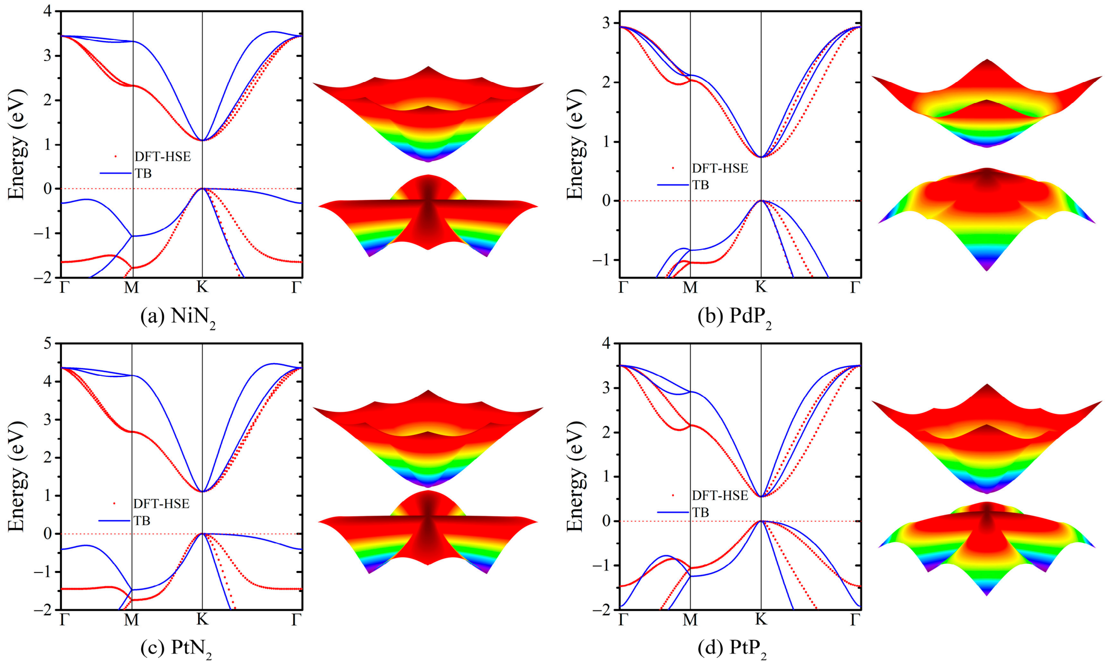

Figure S9. TB band structures of (a) *penta*-NiN_2, (b) *penta*-PdP_2, (c) *penta*-PtN_2, and (d) *penta*-PtP_2 monolayer by fitting the DFT-HES06 band structures. The energy at the VBM is set to zero. Insets are the three-dimensional plots of the highest valence band (VB) and the lowest conduction band (CB) near the Fermi level in the reciprocal space.

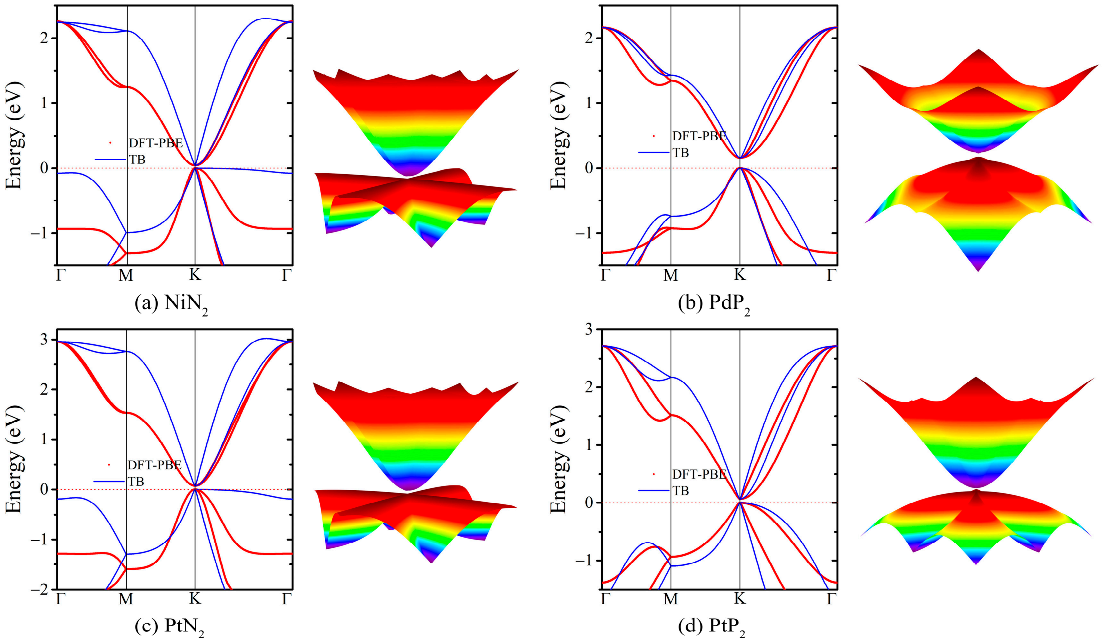

Figure S10. TB band structures of (a) *penta*-NiN_2, (b) *penta*-PdP_2, (c) *penta*-PtN_2, and

(d) *penta*-PtP_2 monolayer by fitting the DFT-PBE band structures. The energy at the VBM is set to zero. Insets are the three-dimensional plots of the highest valence band (VB) and the lowest conduction band (CB) near the Fermi level in the reciprocal space.

S9. Acoustic phonon-limited scattering model and computational details of carrier mobility

An acoustic phonon-limited scattering model was employed to evaluate the carrier mobility, in which the primary mechanism limiting carrier mobility is scattering due to phonons. For a 2D material, the carrier mobility μ_{2D} is determined by the effective mass in the transport direction (m_e^*), the average effective mass (m_d), the deformation potential (E_l), elastic modulus (C_{2D}) and temperature (T) in the experssion [1]:

$$\mu_{\mathrm{2D}} = \frac{e\hbar^3 C_{2D}}{k_B T m_e^* \left(E_l^i\right)^2}.$$

The average effective mass is defined as $m_d = \sqrt{m_x^* m_y^*}$. The deformation potential constant E_l of the VBM for hole or CBM for electron along the transport direction is $E_l^i = \Delta V_i / (\Delta l / l_0)$, where ΔV_i represents the energy change of ith band under proper cell compression and dilatation (calculated using a step of 0.5%), l_0 is the lattice constant in the transport direction and Δl is the deformation of l_0. The elastic modulus C_{2D} of the longitudinal strain in the propagation directions of the longitudinal acoustic wave is derived from $(E - E_0)/S_0 = C(\Delta l / l_0)^2 / 2$, where E is the total energy and S_0 is the lattice volume at equilibrium for a 2D system. The temperature used for the mobility calculations was 300 K.

S10. Light carrier mobility of *penta*-MX_2 monolayer

Carrier Type	[100]				[110]			
	$\frac{m^*}{m_0}$	E_1 (eV)	C_{2D} (J·m^{-2})	μ_{2D} (10^3 cm^2 V^{-1} s^{-1})	$\frac{m^*}{m_0}$	E_1 (eV)	C_{2D} (J·m^{-2})	μ_{2D} (10^3 cm^2 V^{-1} s^{-1})
***Penta*-NiN_2**								
e-light	0.364	2.455	177.544	4.736	0.295	1.300	144.579	21.015
h-light	0.066	0.223	177.544	17361.935	0.036	6.028	144.579	65.036
***Penta*-NiP_2**								
e-light	0.030	1.929	123.837	786.074	0.015	1.045	115.283	10459.342
h-light	0.031	0.562	123.837	8544.841	0.015	1.307	115.283	6687.295
***Penta*-PdP_2**								
e-light	0.189	1.433	115.355	33.495	0.117	0.772	101.284	266.100
h-light	0.102	1.520	115.355	102.595	0.059	5.208	101.284	23.115
***Penta*-PtN_2**								
e-light	0.236	1.791	227.797	27.279	0.169	0.936	175.528	149.175
h-light	0.133	1.260	227.797	173.790	0.086	2.403	175.528	87.957
***Penta*-PtP_2**								
e-light	0.021	1.296	147.023	4305.197	0.011	0.438	134.426	133332.733
h-light	0.021	2.113	147.023	1551.082	0.011	1.348	134.426	13780.329

Table S2. Predicted light carrier mobilities of *penta*-MX_2 monolayer and the relevant parameters along the [100] and [110] directions by employing the PBE functional. m_0 is the mass of a free electron. The unit of carrier mobility is $cm^2\ V^{-1}\ s^{-1}$.

References

1. Xi, J.; Long, M.; Tang, L.; Wang, D.; Shuai, Z., First-principles prediction of charge mobility in carbon and organic nanomaterials. *Nanoscale* **2012,** 4, (15), 4348-4369.
